# Unmasking the mammalian SET domain-containing protein 4

**DOI:** 10.1093/narcan/zcac021

**Published:** 2022-07-13

**Authors:** Yuan Wang, Zhiyuan Shen

**Affiliations:** Rutgers Cancer Institute of New Jersey, Department of Radiation Oncology, Rutgers Robert Wood Johnson Medical School, 195 Little Albany Street, New Brunswick, NJ 08901, USA; Rutgers Cancer Institute of New Jersey, Department of Radiation Oncology, Rutgers Robert Wood Johnson Medical School, 195 Little Albany Street, New Brunswick, NJ 08901, USA

## Abstract

SET domain-containing protein 4 (SETD4) is a member of a unique class of protein lysine methyltransferases. Here, we introduce the basic features of SETD4 and summarize the key findings from recent studies with emphases on its roles in tissue development and tumorigenesis, and its methylation substrates. SETD4 is expressed in stem/progenitor cells. Ablation of Setd4^+^ cells impedes the repopulation of acinar cells after pancreatic injury. *Setd4* deletion in mice promotes the recovery of radiation-induced bone marrow (BM) failure by boosting the function of BM niche, facilitates the generation of endothelial cells and neovascularization of capillary vessels in the heart, enhances the proliferation of BM mesenchymal stem cells and disrupts the TLR4 signaling in BM-derived macrophages. SETD4 expression is also associated with the maintenance of quiescent breast cancer stem cells. While mouse *Setd4* knockout delays radiation-induced T-lymphoma formation, elevated SETD4 expression has been observed in some proliferative cancer cells and is associated with a pro-survival potential. Oncogenic fusions of SETD4 have also been identified in cancer, albeit rare. In addition, SETD4 methylates lysine-570 in the C-terminal globular domain of KU70, which enables KU70 translocation to cytoplasm to suppress apoptosis.

## INTRODUCTION: SET DOMAIN-CONTAINING LYSINE METHYLTRANSFERASE

Protein lysine methylation is a critical post-translational modification that mediates a diverse array of biological processes (1–3). This modification is catalyzed by lysine methyltransferases (4,5) and can be reversed by lysine demethylases (6–8). SET domain-containing proteins harbor a Su(var)3–9–enhancer of zeste–trithorax (SET) domain of ∼130 amino acids (aa) (1,4,5,9,10). A major function of these proteins is to catalyze the transfer of the methyl group from *S*-adenosylmethionine (AdoMet) to the ϵ-amine on the side chain of the lysine residue. While many lysine residues in histones are methylated by SET domain-containing proteins to shape the epigenetic status of cells (1,4,9), a large number of non-histone proteins are also subject to methylation, which modulates their function in various cellular processes (2,3).

The human genome encodes estimated 57 SET domain methyltransferases that can be clustered into 10 classes (11). Class VII of SET proteins represents a distinct cluster of protein methyltransferases that include SETD6, SETD3 and SETD4 (11). SETD6 methylates the p65 (RelA) of the NF-κB family of transcriptional factors (12,13), Polo-like kinase 1 (PLK1) (14), PAK4 of the Wnt/beta-catenin pathway (15) and potentially many more non-histone proteins (16). SETD3 was initially reported to methylate histones (17,18) and be involved in DNA damage response (19). However, independent groups have found that SETD3’s physiological substrate is a histidine residue in β-actin (20–22). The functional features of the third member of this family, SETD4, have been largely unknown until recent years. This review summarizes the basic properties and current studies that have begun to unmask the mysteries of SETD4.

## BASIC FEATURES OF SETD4

**Figure 1.**
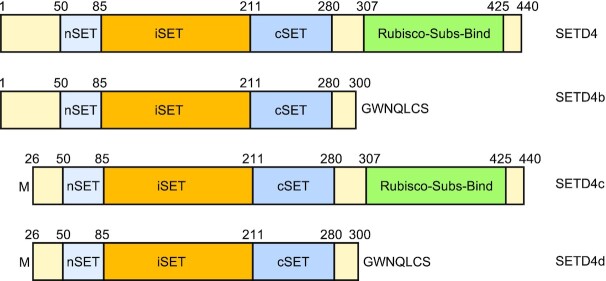
Domain structures of human SETD4. Illustrated are the four curated human SETD4 protein isoforms. All isoforms have the intact SET domain that is split into the nSET and cSET regions by an insert (iSET). The substrate binding domain (Rubisco-Subs-Bind) only exists in SETD4 and SETD4c.

The human *SETD4* gene is located on chromosome 21. It expresses the SETD4 mRNA variant 1 (GenBank NM_017438.5) that encodes the C21ORF18 peptide composed of 440 aa. Due to alternative splicing, at least four more curated mRNA variants of human SETD4 are expressed. They code for three SETD4 protein isoforms (Figure 1) that all contain the SET domain: SETD4b of 307 aa encoded by mRNA variant 2 (GenBank NM_001007259.3); SETD4c of 416 aa encoded by mRNA variant 3 (GenBank NM_001286752.2); and SETD4d of 283 aa encoded by mRNA variant 5 (GenBank NM_001007261.3). However, the SETD4 mRNA variant 4 (GenBank NR_040087.3) is considered a noncoding RNA.

The main isoform of human SETD4 protein has ∼20% of amino acid identity with SETD6 and SETD3. It has a 3D structure similar to those of SETD3 and SETD6, based on predictions using either I-TASSER (23) (https://zhanggroup.org//I-TASSER/) or the AlphaFold Protein Structure Database (24,25) (https://alphafold.ebi.ac.uk/entry/Q9NVD3). The structural features shared by SETD6, SETD3 and SETD4 include (i) a SET domain being interrupted by an iSET region (insert of SET) and (ii) a substrate binding domain with similarity to that of plant Rubisco (ribulose-1,5-bisphosphate carboxylase/oxygenase) large subunit methyltransferase (19,21,22,26). Two tyrosine residues, Y272 and Y284 of human SETD4 (or Y271 and Y283 in mice), are highly conserved among SETD4 proteins from multiple species and are predicted to be part of the activity centers. However, only Y272 is conserved with both SETD3 and SETD6, while Y284 is conserved only with SETD6. Mutation of either Y272 or Y284 alone can dampen the SETD4 activities toward methylating KU70 at K570 (24).

Various SETD4 homologues exist in mammals and other species. For example, the mouse SETD4 protein (439 aa) has ∼78% amino acid identity with almost the entire human SETD4 (aa 24–440), and the brine shrimp *Artemia parthenogenetica* homologue of SETD4 (397 aa) has ∼30% identical residues with an ∼300 aa region of human SETD4 (aa 35–348). Although it is not clear whether there is a SETD4 orthologue in yeasts, the 583 aa *Saccharomyces cerevisiae* RKM1 [ribosomal lysine (K) methyltransferase 1, or YPL208W] has a region of ∼200 aa with 28% identity to human SETD4 (aa 126–296). Interestingly, the yeast RKM1 methylates the large ribosomal subunit protein L23 (27) that interacts with BCP1 (28), which is the mammalian orthologue of BCCIP involved in rRNA processing (29).

Due to these unique features and the mysterious nature of its functions, SETD4 has drawn some attention from several research groups. The observed intracellular localization of SETD4 proteins varied depending on cell types and methods of detection. The human and *A. parthenogenetica* SETD4 (*Ar-SETD4*) were initially reported to be abundant in both nucleus and cytoplasm (30,31). Mouse SETD4 was found predominantly in the cytoplasm of macrophages but could be translocated to the nucleus upon stimulation by lipopolysaccharides (32). However, others have observed SETD4 staining predominantly in the nucleus of quiescent mouse stem cells (33), quiescent cancer stems cells (34) and numerous proliferative cancer cells (24). The discrepancy among observations may reflect the versatile and diverse functions of SETD4 in different physiological contexts.

## ROLES OF SETD4 IN TISSUE REGENERATION AND CELL QUIESCENCE

A role of SETD4 in organ development was first hinted by a study of primordial follicle maturation in mice (35). When a gene-array approach was used to identify ovarian fertility genes (35), mouse *Setd4* was found highly expressed in the ovaries within the first 7 days after birth, and *Setd4* mRNA was enriched in the antral follicles as observed by *in situ* RNA hybridization. However, it was not until *A. parthenogenetica* had been used as a model system that a role of SETD4 in cell quiescence started to be noticed (31). *Artemia parthenogenetica* is a species of brine shrimp with a unique life cycle. When the swimming nauplii are cultured in low-salt condition, *Artemia* females use the ovoviviparous life cycle to directly release swimming nauplii. However, when cultured in high-salt condition, mature *Artemia* females use the oviparous life cycle to release growth-arrested diapause embryos. It was found that the Ar-SETD4 was highly expressed in the cells within the diapause embryos, but its expression was reduced when the diapause embryos were activated to re-enter the proliferation cell cycle. Furthermore, silencing of *Ar-SETD4* in the swimming nauplii abrogated the formation of diapause embryos (31). This study further concluded that Ar-SETD4 expression is required for the embryo arrest, and downregulation of Ar-SETD4 is necessary to relieve the cell cycle arrest allowing the diapause embryos to hatch (31). It represents the first major study that identified a regulatory role of SETD4 in embryo development.

Recently, the Yang lab reported that mouse *Setd4* is also associated with the maintenance of stem and progenitor cells responsible for the generation of the vascular endothelium of capillaries (33), and ablation of *Setd4^+^* cells impedes pancreatic development and repair (36). Using a cell lineage system that tracks the cells with a historical window of SETD4-Cre expression from the endogenous mouse *Setd4* allele, Tian *et al.* reported that *Setd4* is highly expressed in the quiescent progenitor cells that may serve as the reservoirs for pancreatic tissue regeneration (36). Furthermore, selective elimination of *Setd4^+^* cells impeded the pancreatic repair upon cerulein-induced pancreatitis in mice (36). Mouse *Setd4* is highly expressed in the quiescent population of c-Kit^+^ cells isolated from neonatal and adult hearts. Conditional *Setd4* deletion in the c-Kit^+^ cells can trigger the proliferation of quiescent stem/progenitor cells to facilitate neovascularization of capillaries and thereby help preserve cardiac functions in response to injury. These reports suggest a model in which SETD4 expression leads to cell cycle arrest among the quiescent stem or progenitor cells, and downregulation of *SETD4* is required for these cells to re-enter cell cycle to participate in tissue repair, and this may be due to a role of SETD4 in trimethylating H4K20 in these cells (31,33,36).

Mouse SETD4 has been shown to serve as a downstream mediator of the TLR4 signaling pathway in bone marrow (BM)-derived macrophages (32). Interestingly, *Setd4* deletion in adult mice was found to confer a resistance to whole body irradiation that caused BM failure despite enhanced radiation sensitivity of the *Setd4*-null hematopoietic stem cells to radiation (37). While it cannot be ruled out that the *Setd4* deletion directly promoted the entry of survived quiescent hematopoietic stem cells into proliferation, a series of BM transplantation experiments from the same study suggested that *Setd4* deletion can improve the BM niche function after injury (37). This concept is consistent with the observed increase of proliferation of BM mesenchymal stem cells after *Setd4* deletion in mice (38). Together, these reports support a notion that inhibition of SETD4 may contribute to the recovery of BM niche through enhanced proliferation of the mesenchymal cells (37,38). Table 1 summarizes currently reported SETD4 animal models.

**Table 1.** Animal models for SETD4 functions

Species	Genetic approach	SETD4 status	Functions identified	References
*Artemia*	dsRNA injection in diapause-destined adults	Ar-Setd4 KD	Setd4 KD prevented the formation of diapause embryos	(31)
Mouse	Setd4-CreERT2; Rosa26^mT/mG^	Traced Setd4^+^ cells with GFP	Setd4^+^ cells gave rise to acinar cells	(36)
	Setd4-Cre; Rosa26^mT/mG^			
	Setd4-CreERT2; Rosa26^DTA^	Ablation of Setd4^+^ cells	Ablation of Setd4^+^ cells compromised the regeneration of acinar cells	
	Setd4-CreERT2; Rosa26^mT/mG^	Traced Setd4^+^ cells with GFP	Setd4 is expressed in quiescent c-Kit^+^ cells	(33)
	c-Kit-CreERT2; Setd4^flox/flox^; Rosa26-TdTomato	Setd4 KO in c-Kit (red)^+^ cells	Setd4 KO increased vascular endothelial cells	
	Setd4^flox/flox^; Rosa26-CreERT2	Setd4 KO	Setd4 KO resulted in a delay of radiation-induced thymic lymphoma and improved recovery of hematopoietic failure	(37,46)
	Setd4^flox/flox^; EIIa-Cre	Setd4 KO	Setd4 KO in BMDMs reduced expression of inflammatory cytokines	(32)
	Setd4^−/−^	Setd4 KO	Setd4 KO promoted the proliferation of BMSCs	(38)
	Setd4 wt; Ku70^K568R/K568R^	SETD4 cannot methylate KU70 due to K568R mutation	Setd4 suppresses apoptosis by methylating KU70	(24)

KD: knockdown; KO: knockout; BMSCs: bone marrow mesenchymal stem cells; BMDMs: bone marrow-derived macrophages.

**Figure 2.**
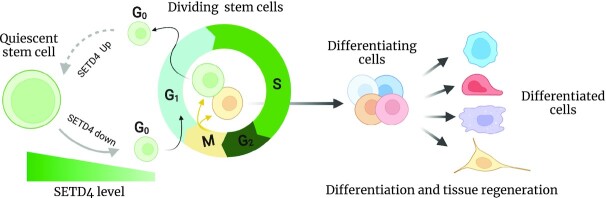
Association between SETD4 expression and quiescent stem cell population. Shown is a model illustrating the potential role of SETD4 in maintaining the quiescent stem cell pool. SETD4 is highly expressed in the quiescent stem/progenitor cells. Upon stimulation, SETD4 level declines and the quiescent stem cells enter the cell cycle to produce proliferative cells.

Collectively, the studies with diapause embryos of *A. parthenogenetica* (31), damaged mouse BM (37), injured pancreas (36), vascular endothelium (33) and BM mesenchymal stem cells (38) have revealed a shared role of SETD4 in maintaining the stem cell population during development and organ regeneration and repair. As modeled in Figure 2, SETD4 expression may serve as a barrier to prevent quiescent cell/progenitor cells from re-entering the proliferation cell cycle, and thus help maintain a pool of quiescent stem/progenitor cells. A programmed reduction of SETD4 expression may allow the quiescent stem/progenitor cells to re-enter cell cycle and promote tissue repair. This model predicts that a persistent SETD4 loss may result in a premature exhaustion of stem cell population, which would need to be tested in the future.

## ROLES OF SETD4 IN TUMORIGENESIS

**Figure 3.**
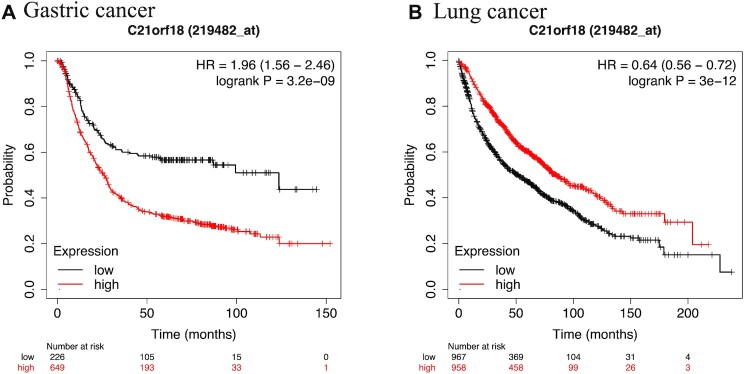
Association of SETD4 expression with the survival rates of gastric and lung cancer. An online database (https://kmplot.com/analysis/index.php?p=service) was used to stratify the association of SETD4 (C21orf18) expression with the survival outcomes of gastric cancer (**A**) and lung cancer (**B**). The methods of database construction and analyses are described in refs. (44,45). Red curves represent the survival rates of cancer with top 50% of SETD4 expression, and black curves represent the survival rates of cancer with the lower 50% of SETD4 expression. The *P*-value of logrank test is displayed in the upper right corner in each panel.

Several early reports had implicated SETD4 expression in promoting growth and drug resistance of cancer cells. SETD4 overexpression was suggested to associate with estrogen receptor-positive breast cancer, albeit a limited number of cell lines and tumor tissues were analyzed (30). RNAi screens identified SETD4 as one of the methyltransferases whose silencing enhanced the sensitivity of hepatocellular carcinoma cell line HepG2 to sorafenib (39) and affected the viability of the androgen-independent prostate cancer cell line DU145 (40). Furthermore, genomic studies indicated an oncogenic potential of SETD4 fusions. According to the TCGA database and two reports (41,42), *SETD4* was fused with *TMPRSS2* and *ERG* in prostate cancers, with *FTCD* in invasive ductal carcinoma, with KIAA1958 in serous ovarian cancer and with B4Galt6 in lung squamous cancer. The *SETD4* fusions with *TMPRSS2*, *ERG* and *FTCD* were likely caused by intrachromosomal deletions of chromosome 21. However, the fusions with *B4GALT6* (on chromosome 18) and *KIAA1958* (on chromosome 9) were likely caused by interchromosomal translocations. Most recently, SETD4 mRNA was found to be stabilized by a long noncoding RNA CBR3-AS1 in gestational choriocarcinoma, and the subsequent SETD4 overexpression may be responsible for the tumor growth in CBR3-AS1-expressing tumor (43). As shown in Figure 3, gene expression analyses using Affymetrix array suggested that high levels of *SETD4* expression were associated with worse survival rates for gastric cancer (44), but better survival rates for lung cancer (45). Thus, the association between *SETD4* expression and the outcome of cancer treatment would need to be assessed in a cancer type-specific manner.

Although the aforementioned studies have hinted at an involvement of SETD4 in tumorigenesis, SETD4’s direct role in tumorigenesis has largely been elusive until three recent studies that were conducted with quiescent breast cancer stem cells (qBCSCs) (34), with a mouse model of radiation-induced T lymphoma (46) and with a newly identified non-histone substrate of SETD4 (24), respectively. Based on the expression of CD44^high^/CD24^low^ and PKH26^+^, the Yang lab reported that a subpopulation of MCF-7 and other BCSCs also had high levels of SETD4. This subpopulation of BCSCs was quiescent and thus termed qBCSCs. Interestingly, SETD4 mRNA was highly expressed in the qBCSC population. A stimulation of qBCSCs to re-entry into cell cycle was associated with SETD4 downregulation. Furthermore, exogenous expression of SETD4 suppressed the tumorsphere formation from the BCSCs (CD44^high^/CD24^low^), whereas knockdown of the exogenous SETD4 restored tumorsphere formation. This report further suggested that trimethylation of H4K20 by SETD4 might be epigenetically responsible for the quiescent status of the qBCSCs (34). These results were consistent with an earlier report from the same lab (31), which demonstrated that Ar-SETD4 expression is required for cell cycle arrest in diapause embryos of *A. parthenogenetica*.

Using a radiation-induced T lymphomagenesis model, Feng *et al.* reported that an induced *Setd4* deletion in adult mice delayed T-lymphoma formation (46). Interestingly, the delayed time course of T lymphomagenesis in the *Setd4* knockout mice was highly similar to the results with *Tlr4* knockout mice (47), which is in agreement with the suggested role of SETD4 as a downstream mediator of TLR4 signaling (32).

Most recently, Wang *et al.* have demonstrated that SETD4 is highly expressed in some proliferative cancer cells, such as H1299 and A549, but modestly expressed in others, such as HT1080 and U2OS (24). SETD4 can methylate human KU70 at residue K570 (equivalent to mouse K568) and confers a resistance to apoptosis induction (24). Although KU70 has a well-established role in the repair of DNA double-strand breaks, this study revealed that KU70-K570 methylation by SETD4 enables the KU70 to translocate to the cytoplasm and may serve as a nucleus-to-cytoplasmic signal mediator to coordinate DNA repair and antiapoptotic events in cells. The newly identified role of SETD4 in mediating KU70-dependent suppression of apoptosis is also in agreement with the observed drug sensitization of HepG2 upon SETD4 silencing (39). Table 2 summarizes the reported SETD4 expression status in cell lines.

**Table 2.** Relative level of SETD4 expression and localization reported in different cell lines

		Expression				
Host	Cell line	mRNA	Protein	Antibody used	Intracellular localization	Reference
Human	HCC-1954-BL	+	N/A	Santa Cruz (cat# N/A)	Nuclear/cytoplasm	(30)
	HCC-1954	+++	N/A			
	MCF-7	+	+			
	CAMA-1	+	N/A			
	SKBR 3	+	N/A			
	MDA-MB 231	++	N/A			
	MDA-MB 436	++	N/A			
	MDA-MB 468	+	N/A			
	MACL-1	N/A	++			
	MGSO3	N/A	++			
	MDA-MB 231	N/A	++			
Human	MCF-7-qBCSCs	N/A	+	Sigma (cat# HPA035405, cat# HPA024073) Santa Cruz (cat# sc-514060)	Nuclear	(34)
	HCC1937-qBCSCs	N/A	+			
Mouse	RAW264.7	+	+	Santa Cruz (cat# sc-133993, cat# sc-83749)	Nucleus/cytoplasm	(32)
Human	HT1080	N/A	+	Lab generated, h25 (cat# N/A)	Mostly nuclear	(24)
	U2OS	N/A	+			
	HepG2	N/A	++			
	H1299	N/A	+++			
	A549	N/A	++			
	MCF-7	N/A	++			
	HeLa	N/A	+			
	293	N/A	+			

In summary, newly emerging evidence suggests that altered SETD4 activities may contribute to different aspects of tumorigenesis and mediate tumor response to therapy. However, it should be cautioned that the specific consequence of SETD4 alterations could be significantly influenced by the biological context of the specific tumor. More in-depth studies are much needed to explore the therapeutic potential of targeting SETD4 in cancer intervention.

## METHYLATION SUBSTRATES OF SETD4

### Histone substrates of SETD4

To date, a full spectrum of SETD4 substrates is not yet available due to the lack of systematic or proteomic approaches. However, several targeted studies have suggested a histone methyltransferase activity of SETD4. These conclusions were mostly based on monitoring the methylation levels of selected histones upon increased or downregulated expression of SETD4, or *in vitro* methylation assays using purified histones and recombinant SETD4. In the first report addressing the SETD4’s histone substrates, it was shown that, among ∼18 methylation forms of H3K4, H3K9, H3K27, H3K36, H3K79 and H4K20, only H4K20me3 and H3K79me3 levels were reduced upon Ar-SETD4 knockdown in *A. parthenogenetica*, while H4K20me3 and H3K79me3 levels were enhanced upon Ar-SETD4 overexpression in cells (31). However, only H4K20me3 was produced by *in vitro* methylation assay using recombinant Ar-SETD4 protein. In a second study from the same laboratory, it was shown that exogenous expression of mammalian SETD4 enhanced the level of H4K20me3 in MCF-7 and HCC1937 cancer cells, and purified mammalian SETD4 produced H4K20me3 *in vitro*. H4K20me3 is known to deposit on constitutive heterochromatin such as the pericentromeric, telomeric or ribosomal regions of chromosomes (48). SETD4 expression in the qBCSCs promoted an enriched deposition of H4K20me3 in the promoters of key genes regulating cell proliferation (34). These studies suggested a role of SETD4 in maintaining the quiescent status of stem cells via H4K20me3, which has been supported by a later report from the same lab (33). However, the level of H4K20me3 was found to be unaffected by SETD4 in BM-derived macrophages during inflammatory response (32). In another report with BM mesenchymal stem cells, SETD4 knockdown also failed to induce H4K20me3 despite its induction of mono- and dimethylations of H4K20, H3K4, H3K36 and H3K79 (38). Thus, it is possible that SETD4 has the potential to methylate multiple histone substrates, while different substrate(s) may be activated dependent upon cell types and biological context. Table 3 summarizes the currently reported methylation products by SETD4.

**Table 3.** SETD4 protein purification and substrates/products identified *in vitro* and *in vivo*

*In vitro* (recombinant protein)				In cells			
Host	Tagged SETD4	Purification source	Methylation products	Host species	Cell line	Methylation products	Reference
*Artemia*	GST-Ar-SETD4	*Escherichia coli*	H3K79me3 H4K20me3	*Artemia*	Human MKN45 (O/E)	H3K79me3 H4K20me3	(31)
Mouse	His-SETD4	293	H3K4me1 H3K4me2	Mouse	BMDMs	H3K4me1 H3K4me2	(32)
–	–	–	–	Mouse	BMSCs	H4K20me1 H4K20me2 H3K4me1 H3K4me2 H3K27me2 H3K36me1 H3K36me2 H3K79me1 H3K79me2	(38)
–	–	–	–	Mouse	c-Kit^+^ cells	H4K20me3	(33)
Human	MBP-SETD4	*E. coli*	KU70K570me1 KU70K570me2 KU70K570me3	Human	HT1080 (O/E) U2OS (O/E) H1299 A549	KU70K570me3 (major) KU70K570me2 (minor)	(24)

O/E: overexpression; BMDMs: bone marrow-derived macrophages; BMSCs: bone marrow mesenchymal stem cells.

### KU70 as a non-histone substrate of SETD4

**Figure 4.**
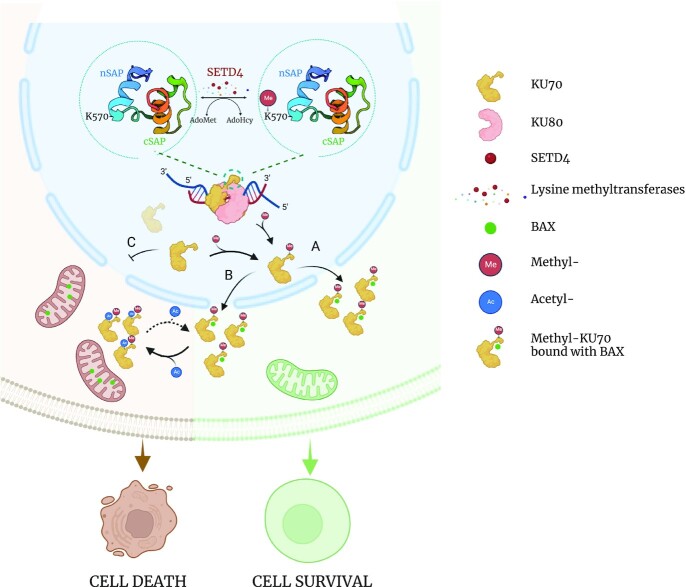
A model of cell fate determination as regulated by post-translational modifications of the KU70 C-terminal SAP domain. This model was mainly based on data from two published works (24,50). The C-terminal 71 amino acids (aa 539–609) of KU70 consist of a linker region (aa 539–559), the KU70-specific nSAP (aa 560–572) and the canonical cSAP (aa 573–609). Residue K570 within the nSAP is subject to SETD4-mediated methylations, with trimethylation being the dominant form. While it has not been determined whether the methylation occurs when KU70 is in dimer form with KU80 and/or as the monomer, the reaction likely occurs predominantly in the nucleus. The combined consequence of KU70 methylation and acetylation can offer three cell fate options. In scenario A, upon K570 methylation, KU70 can be translocated to cytoplasm and bind to cytosolic BAX to sequester it from entering mitochondria and promote cell survival. In scenario B, KU70 is methylated and translocated to the cytosol to temporally block BAX, but can be subjected to acetylation in the linker region (50), which results in the release of BAX from KU70 and leads to BAX-mediated cell death. In scenario B, deacetylation of KU70 may reverse the course of apoptosis. In scenario C, defective KU70 methylation precludes KU70 from entering cytosol; thus, cytosolic BAX cannot be sequestered, and apoptosis is unrestrained.

Although most of the SET domain proteins have documented lysine-methylating activities on histones, bioinformatics and structural modeling approaches have suggested that class VII of SET proteins, including SETD6, SETD3 and SETD4, represents a distinct cluster of non-histone methyltransferases. As an attempt to determine other substrates of SETD4, we screened for SETD4-interacting proteins and identified KU70. The human KU70 C-terminus has a globular domain (aa 559–609), often referred as KU70-SAP domain. In addition to the originally defined SAP (SAF-A/B, acinus and PIAS) motif (49), termed as KU70-cSAP (aa 573–609), the KU70-SAP contains an extra helical domain (aa 559–572) termed as KU70-nSAP. Based on the published structure of KU70-SAP and a modeled SETD4 structure, we predicted and then experimentally verified that lysine-570 in the KU70-nSAP is a SETD4 substrate. The conclusion of KU70-K570 methylation was based on *in vitro* observation of KU70 methylation by SETD4, a reduction of KU70 methylation upon SETD4 downregulation in tumor cells with high levels of SETD4 and an induction of KU70 methylation due to exogenous expression of SETD4 in cells with modest endogenous SETD4 (24). While nuclear KU70 dimerizes with KU80 in repair of DNA double-strand breaks, our results suggested that the methylation of KU70 leads to its disassociation from KU80 and translocation to the cytoplasm where KU70 functions as a suppressor of apoptosis (Figure 4, scenario A). It is noteworthy that the methylation of KU70 by SETD4 likely occurs in the nucleus and methylated KU70 is then translocated to the cytoplasm, where it can be subjected to further regulation by acetylation (Figure 4, scenario B). This suggests that methylation of KU70-K570 is a prerequisite for the cytoplasmic acetylation of KU70, since unmethylated KU70 cannot be enriched in the cytoplasm (Figure 4, scenario C). This study raises a possibility that the methylation of KU70 in the nucleus may serve as a messenger to the cytoplasm to promote cell survival. The identification of KU70 as a substrate unmasks a new role of SETD4 in methylating non-histone protein(s), moving us a step closer to a full understanding of SETD4.

## PERSPECTIVES AND CONCLUDING REMARKS

After the annotation of C21ORF18, the recognition of its similarity to SET proteins and subsequent nomenclature of SETD4, there had been limited understanding on its functions until recent years. The new studies reviewed herein have suggested mammalian SETD4 expression in specific cell types and its role in maintaining the quiescent population of stem/progenitor cells. Although these works have unmasked some features of SETD4, including its enzymatic activity toward both histone and non-histone proteins, additional questions remain to be addressed to grasp the full picture of SETD4’s role in development and tumorigenesis.

It remains an open question whether other histone or non-histone substrates exist under physiological conditions, although KU70 is the only protein confirmed to interact with SETD4 in our yeast two-hybrid system screens. The efforts to identify the full substrate spectrum and to perform comprehensive analysis of its enzymatic activity have been hampered by several practical obstacles. For example, the *in vivo* approaches are hindered by the unreliable enrichment of methylated proteins, a lack of cell system that expresses physiological levels of SETD4 in a large cell population, while the *in vitro* assays are impeded by the challenge of obtaining a large amount of undegraded and soluble recombinant SETD4 protein. In addition to learning the full list of SETD4 substrates, it would also be important to understand whether a SETD4 substrate may be redundantly methylated by other SET domain proteins. For example, H4K20 is known to be trimethylated by SETD8 to maintain the constitutive heterochromatin and genomic integrity (48); thus, a question remains whether SETD4 interfaces with SETD8.

The significance of the putative human SETD4 isoforms remains to be determined. It has been noted that all human SETD4 isoforms have the intact SET domain (Figure 1). Thus, one may wonder whether they have distinct substrate preferences if expressed in different cell types. It would also be interesting to know whether the specific expression of isoforms may affect cell type-specific functions. In addition, it is unclear whether the long noncoding RNA variant of SETD4 bears any biological function in cells.

Lastly, in stem/progenitor and some cancer cells, SETD4 expression confers a quiescent status, likely due to an enhanced H4K20me3 status as previously proposed (31,34,36). However, some dividing cancer cells clearly have an elevated SETD4 expression but are proliferative and do not seem to be growth arrested (24). The apparent counterintuitive functions of SETD4 in quiescent cancer stem cells and proliferative cancer cells may hint at a synthetic viability mechanism by which SETD4-mediated growth arrest is bypassed in the proliferative cancer cells. It is essential to have a more thorough understanding of the dynamics of SETD4 expression in different cell types during development and tumorigenesis, and to identify the authentic substrates in each of the cell types.
